# What are the advancements in surgery?

**DOI:** 10.1097/JS9.0000000000000533

**Published:** 2023-06-07

**Authors:** Jie Huang

**Affiliations:** Department of Hepatopancreatobiliary Surgery, The Second Affiliated Hospital of Kunming Medical University, Kunming, Yunnan, People’s Republic of China

*Dear Editor*,

On the eve of his retirement, Japanese surgeon Prof Masatoshi Makuuchi stated ‘The question is how the “progress” in the field of surgery be presented. It should be presented as a collection of clinical achievements that deserve to be published in refereed international academic journals’. This has been facilitated through the tireless efforts of numerous pancreatic surgeons who have enthusiastically shared their experiences with laparoscopic pancreatic surgery using different mediums, such as literature and videos, thereby enabling more patients to benefit from laparoscopic technology.

With immense interest, I read the article by van Ramshorst *et al*.^1^, which was recently published in the *International Journal of Surgery*. van Ramshorst *et al*. compared the learning curves and outcomes of laparoscopic distal pancreatectomy (LDP) between the ‘self-taught’ and ‘trained’ surgeons in terms of feasibility and proficiency using short-term outcomes. They concluded that, compared with self-taught surgeons, trained surgeons demonstrated at least a 50% reduction in the feasibility and proficiency learning curves for LDP. Yet, as a trained surgeon, I have the utmost respect for self-taught surgeons.

Laparoscopic surgery is a highly complex surgical technique that demands specialised training and expertise for mastery. However, there are instances where surgeons must perform laparoscopic surgery without prior training. The first LDP was reported in 1994^2,3^; we can only imagine the execution of LDP by our predecessors during that era.

The visual field differs during laparotomy and laparoscopy. The former provides a ventral-to-dorsal view, whereas the latter offers a foot-to-dorsal view. Although several simulation training courses are available for self-taught surgeons, attempting an independent LDP can pose unexpected challenges. First, self-taught surgeons experience difficulties in selecting the appropriate cases during their learning curve because of the stark contrast between the visual field of laparotomy and endoscopy, with the latter providing magnification. Additionally, some self-taught surgeons may overlook moral anatomy prior to laparotomy, necessitating more time for dissection and identification. Consequently, reaching the inflection points of the feasibility learning curve requires a longer duration. In this paper, the author also reported that self-taught surgeons encounter more inflection points in surgical cases, thereby encountering more complex situations during the surgical process because of their lack of prior experience.

Furthermore, laparoscopic surgery emphasises teamwork. The entire team must work cohesively in various aspects, including patient positioning, intra-operative anaesthesia management, surgical procedures and judicious use of instruments during the surgery. Most of the trained surgeons worked as assistants with self-taught surgeons to accomplish multiple operations, but they do not perform these surgeries independently. In China, we often address these surgeons as the 2.0 generation of laparoscopic surgeons, and I belong to this category of surgeons too. Prior to performing laparoscopic pancreatic surgeries independently, I had assisted self-taught surgeons in performing numerous complex surgeries and gained extensive surgical experience while handling different cases. My past experience as an assistant allowed me to quickly clear the learning curve when I independently performed laparoscopic pancreatic surgery. Likewise, when I was training the younger generation of laparoscopic surgeons, they were able to gain vast surgical experience and skills through live surgical broadcasts and video articles in journals. Would this not be considered progress in the field of surgery? It is possible that laparoscopic 3.0 surgeons may experience a shorter learning curve to achieve technical stability, which would represent a considerable improvement in surgical practice.

I would like to express my gratitude to Tess M.E. van Ramshorst *et al*. for their research, which not only showcases the remarkable achievements of laparoscopic surgery pioneers but also sets a higher standard for future generations of laparoscopic surgeons. Thank you for your invaluable contribution.

## Ethical approval

This paper is a correspondence and does not require ethical approval.

## Consent

This paper is a correspondence and does not require consent.

## Sources of funding

This work was supported by the Yunnan Fundamental Research Projects (Grant No. 202101AT070239), and the Investigator Initiated Trail Projects of the Second Affiliated Hospital of Kunming Medical University (Grant No. 2020ynlc004).

## Author contribution

J.H.: writing and editing and supervision.

## Conflicts of interest disclosure

No potential financial and non-financial conflicts of interest.

## Research registration unique identifying number (UIN)

No.

## Guarantor

Jie Huang.

## Data availability statement

No.

## Provenance and peer review

No.
